# Vitamin D supplementation improves FEV1 in patients of Bronchial Asthma

**DOI:** 10.12669/pjms.335.12990

**Published:** 2017

**Authors:** Muhammad Zafar Majeed Babar, Mazhar Hussain, Sadia Abdul Majeed

**Affiliations:** 1 Muhammad Zafar Majeed Babar, FCPS Medicine. Associate Professor of Medicine, Sheikh Zayed Medical College/Hospital, Rahim Yar Khan, Pakistan; 2 Mazhar Hussain, M. Phil Pharmacology. Assistant Professor of Pharmacology, Sheikh Zayed Medical College/Hospital, Rahim Yar Khan, Pakistan; 3 Sadia Abdul Majeed, MBBS. Demonstrator Anatomy, Sheikh Zayed Medical College/Hospital, Rahim Yar Khan, Pakistan

**Keywords:** Bronchial asthma, FEV1, vitamin D

## Abstract

**Background and Objective:**

Vitamin D deficiency has strong association with various respiratory disorders in which bronchial asthma is one of them. The objective was to determine the efficacy of vitamin D supplementation in cases with bronchial asthma.

**Methods:**

This case control study was conducted at private clinical set up of district, Rahim Yar Khan from August to October 2016 in which 100 cases of bronchial asthma were randomly divided into Group-A and Group-B each contained 50 patients. Group-A was given placebo and Group-B with vitamin D in a dose of 50,000 units per day orally. Both the groups were followed in terms of improvement in FEV1 at 1, 2 and 3 months.

**Results:**

There was no significant difference in both groups in terms of BMI and duration of asthma at start of study. The mean pre treatment vitamin D level of Group-A was 14.23±1.66 and of Group-B, 15.30±2.05 ng/dl (p= 0.23). FEV1 in pre treatment Group-A was 64.35±3.16 and of Group-B was 62.35±2.16 with p= 0.95. There was no significant difference in terms of FEV1 in both the groups at one month (p= 0.32). While at two months it was significantly higher in Group-B with p= 0.04. At 3 months the final outcome was seen where the post treatment FEV1 in Group-A was 66.13±2.75 and in Group-B, 75.15±2.04 with p value of 0.001.

**Conclusion:**

Vitamin D supplementation improves FEV1 significantly at two months and these can be even highly significant if it is extended up to 3 months.

## INTRODUCTION

Asthma is one of the major health issues across the world from childhood to adult life. According to a survey more than 25 million people are suffering from this in United States in 2012 and is one of the most reported entity in both outpatient and inpatient departments. It posed a great burden in terms of economy. The role of environmental factors in the prevalence of asthma cannot be denied but recent researches have focused more on metabolic and nutritional factors in which vitamin D level is one of them. Despite all the advances in the medical sciences and researches there are no definitive treatment to eradicate it and the options used are to control it and sometimes fail to do it, for which newer agents are always in need.^1^,^2^

Vitamin D is one of the newer agents that are being studied in the treatment of such cases. Vitamin D has been thought to act in various ways. Alpha hydroxylase is present in the lung epithelium which is capable of converting calcidiol to calcitriol; and this is thought to interfere in the synthesis of different cytokine i.e. RANTES, PDGF, interleukin, different CD cells and metalloproteinases. These are the markers of inflammation found in the cases with asthma and enhance the inflammatory mechanisms. Vitamin D is presumed to decrease in the infection rate as well due to cathelcidine, which is over expressed with its supplementation and is anti infective in nature.^3^,^4^

Extensive work up is being done in recent times to build the association of its level with various respiratory conditions and in many circumstances it is also proved that the low levels of 25-hydroxy-vitamin D are associated with increased respiratory tract infection, decreased lung function tests, reduced steroid response and frequent exacerbation.^5^-^8^

The present study was designed to see the effect of vitamin D supplementation in the improvement of symptoms of bronchial asthma in terms of FEV1.

## METHODS

This case control study was conducted at private clinical set up of district, Rahim Yar Khan from August to October 2016 in which 100 cases of bronchial asthma aged 18 to 50 years were randomly divided into Group-A and Group-B each contained 50 patients. The diagnosis of bronchial asthma was made by PFTs with FEV1/FVC ratio of 0.7 and reversibility in FEV 1 of more than 12% in cases with history of cough, shortness of breath or chest tightness. The cases with history of renal, liver disease, pregnancy were excluded from the study. The demographics like age, BMI, duration of asthma and baseline level of vitamin D was checked and data was collected. The cases were asked to select one of the shield opaque envelope labeled as A or B. The cases in Group-A were treated as per GINA guidelines 2016 according to severity of disease along with placebo and Group-B was also treated with anti-asthmatic drugs as per severity and vitamin D in a dose of 50,000 units per day orally. Both the Groups were followed in terms of FEV1 at one month, two months and three months where final outcome was seen. Efficacy was assessed in-terms of improvement in FEV1.

### Statistical Analysis

A sample of 50 cases in each group was selected. Both the groups were compared in terms of their mean age, BMI, vitamin D level, baseline FEV1. Data was stratified to see for significance in both groups regarding efficacy. Independent sample t-test and chi square test were used to compare the both groups taking p-value of ≤ 0.05 as significant.

## RESULTS

There were total 100 cases, 50 in each group. Group-A contained 26 (52%) males and 24 (48%) females, and Group-B has 29 (58%) males and 21 (42%) females (Table-I). The mean age of Group-A was 26.50±5.50 and Group-B, 27.60± 6.50 years with p=1.04. There was no significant difference in both groups in terms of BMI and duration of asthma. The mean pre treatment vitamin D level of Group-A was 14.23±1.66 and of Group-B, 15.30±2.05 ng/dl (p= 0.23). FEV1 in pre treatment Group-A was 64.35±3.16 and of Group-B was 62.35±2.16 with p= 0.95 as in Table-I. There was no significant difference in terms of FEV1 in both the groups at one month (p= 0.32). While at two months it was significantly higher in Group-B with p= 0.04. At three months the final outcome was seen where the post treatment FEV1 in Group-A was 66.13±2.75 and in Group-B, 75.15±2.04 with p value of 0.001 as in Table-II.

**Table-I:** Comparison between variables of two groups.

*Variable*	*Group-A*	*Group-B*	*p-value*
Male/Female	26/24	29/21	0.87
Age (years)	26.50±5.50	27.60± 6.50	1.04
BMI (Kg)	24.75±3.20	26.70±6.40	0.96
Duration of asthma	12.40±3.50	11.66±3.05	0.66
Pre-treatment vit D level	14.23±1.66	15.30±2.05	0.23
Pre treatment FEV 1	64.35±3.16	62.35±2.16	0.95

**Table-II:** Efficacy comparison between two groups.

*FEVI 1*	*Group-A*	*Group-B*	*Significance*
FEV 1 at baseline	64.35±3.16	62.35±2.16	p= 0.95
FEV 1 at 1 month	65.65±3.05	66.28±1.10	p= 0.32
FEV 1 at 2 months	65.84±2.95	71.44±2.78	p= 0.04
FEV 1 at 3 months	66.13±2.75	75.15±2.04	p= 0.001

## DISCUSSION

Bronchial asthma is a high burden disease in developing countries like Pakistan and their management relies upon the pharmacotherapies for which new guidelines are recommended every year by NICE group and are tried to meet. But in some cases even with these controller and reliever medications, there are cases, which are still not responding to conventional treatments. This again calls for looking for the other factors, which can be associated with these, as asthma is multifactorial and different pathophysiological mechanisms are involved with different degrees of severity in individual cases. Recent data also support that the current treatment options used in the treatment of asthma are not enough and newer and better options are required.

In this study there was significant difference in terms of FEV1 in both the groups at two months with p= 0.04. At three months the final outcome was seen where the post treatment FEV1 in Group-A 66.13±2.75 and in Group-B, 75.15±2.04 with p value of 0.001. This was also proved by other studies as well that the asthma symptoms were improved with treatment of vitamin D supplementation. In a study done by Black and Scragg et al., they found strong association in improvement of asthma symptoms in term of FEV 1 and improvement in FVC with vitamin D3.^9^ While in recent study done by Gargen et al. in 2013, they used vitamin D supplementation and reported that the cases treated with this have lower chance of respiratory tract infections especially allergic conditions and also revealed that the higher the concentration of vitamin D and lower are the levels seen of IgE level and peripheral eosinophilia.^10^ This again re enforces its use in cases of asthma considering it as enhanced allergic phenomenon and those cases where there is increased eosinophil and IgE level. Bergman et al also report decreased incidence of respiratory infections in vitamin D augmented group.^11^ However, studies done by Iqbal et al. also reported improvement in inflammation in cases with asthma, however they did not find any significant association with this.^12^

In a study done by Dupont et al.^13^ they added leukotriene receptor blocker with vitamin D and study done by Korn et al., Shah et al and Keith et al also used it as an add on and revealed improvement in cases with asthma.^14^-^16^ However they studies these population for 8 weeks at which we also found significant improvement with p-value of 0.04 but at 3 months we found even more significant improvement with p= 0.001

## CONCLUSION

Vitamin D supplementation improves FEV1 significantly at eight weeks and these can be even highly significant if it is extended up to three months.

### Authors` Contribution

**MZMB:** Conceived, designed the methodology, Supervised the research project and gave final approval for publication.

**MH:** Manuscript writing and helped in statistical analysis.

**SAM:** Helped in statistical analysis and did review.
